# Toxemia and Infections as Causes of Insanity1Read at the 37th annual meeting of the Medical Association of Central New York, held at Rochester, N. Y., September 20, 1904.

**Published:** 1905-01

**Authors:** Frank Stephenson

**Affiliations:** Syracuse, N. Y.


### Toxemia and Infections as Causes of Insanity.[1]

By FRANK STEPHENSON, M. D., Syracuse, N. Y.

REGARDING the etiology of insanity, it is of comparatively recent date that particular attention has been directed toward the effect upon the brain of toxins developed by fermentation and putrefaction in the gastrointestinal tract,—faulty metabolism,—and from the poisons in the blood developing from acute infections. According to Peterson "these toxin producing bacteria may be the chief agents whose influence acts upon the cortical cells and nerve fibers of the brain." It is not strange that the blood charged with these toxins should carry agents to the brain, which cause a disturbance of its functions, as we know the blood is the source of all the brain's nutrition and the channel for exit of its waste material.

In brain disorders, even without any history having been given of disturbed digestion, on investigation we find evidences of extensive digestive and fermentative changes shown by urinary analysis; that is, diminished or increased specific gravity, abnormal color, a lack of proper ratio between the specific gravity and the amount of urea eliminated; the presence of indican, zanthins, urates, phosphates, oxalates, and other products. Wagner, in the *Klinische Wochenschrift,* February, 1903, reports several mental cases in which indican and acetonuria were in excess, and that some of these cases, characterised by severe depression left

---

1. Read at the 37th annual meeting of the Medical Association of Central New York, held at Rochester, N. Y., September 20, 1904.

his clinic cured after three days of thorough eliminative and anti-fermentative treatment. The correction of these errors is also the first step in producing sleep, and in quieting the various nervous and mental manifestations in both borderline and classified insanities. We also prepare the system for making more and better blood, thus controlling not only the toxemia but the anemia which is sure to develop and be a stubborn factor in all of these patients.

F. Mott (*London Lancet*, August, 1901,) says, "the new neurology does not involve merely an acceptance of the neuron theory, but a recognition of the fact that every cell possesses a biochemical sensitiveness to its lymph environment, termed chemotaxis; the alteration of the environment causing, in many cases, the presence of some form of poison." And the essential cause of a class of diseases of the nervous system, which includes functional disorders and primary degenerations, is the failure of the neuron to carry on the processes of assimilation and dissimilation, which are essential for the well-being of every cell." We believe, also, that poisons circulating in the blood act as contributory, predisposing or exciting causes, in persons with a neuropathic or psychopathic heredity, and in persons who have subjected their nervous systems to excessive functional activity. Toxins may either be introduced into the body from without or they may be produced within,—exogenetic or endogenetic. In either case, however, it is the toxic state of the blood or lymph which causes the morbid nervous phenomena, but the symptoms which arise depend upon conditions of either increased excitability or depressed excitability of the nervous elements which are selected by the poison.

Franz Glenard (*Le Progrés Medical*, March, 1902,) says, "there is undoubtedly an enteroptosic neurosis, a hepatic neurasthenia and a hepatic agoraphobia which require for treatment regulation of the diet, and laxatives, instead of bromides, hydrotherapy and tonics. In every neurosis or psychosis the digestive apparatus should be explored, including the liver, intestine, stomach, kidney and spleen, as to their size, consistency, sensitiveness, location, relation and degree of fixity." Macpherson, at the Stirling insane asylum, says he submits all such patients to lavage of the stomach, laxatives, intestinal antisepsis and strict diet. He reports the case of a young man of sedentary habits, who was attacked with neurasthenic anxiety and digestive cachexia with vomiting, and was so ill it was feared he would die. He recognised nobody and talked as if insane, but after purgation and vomiting excessively he became rational, slept well, and the next morning was in his usual health.

All of my acute or incipient cases of mental disease, I have treated along these lines for some years. Many of these patients are able to come to the office where lavage can be given, and taught them or members of the family if a nurse is not in charge. Frequent prolonged hot baths act as a most excellent sedative and favor elimination, which is so beneficial to these patients. I insist upon their drinking eight glasses of water daily, hot or cold, and insist upon their taking much exercise in the open air if strong enough to do so, and if the urinary analysis, blood tests, general symptoms and condition seem to require it, I place many upon the following prescriptions:

Tan albumen in 15 gr. doses after meals.
Calomel, 1 gr. at night.

These arrest fermentation and overcome putrefactive changes.

Dilute hydrochloric or phosphoric acid also produces good results in some of these cases, but the following seem to me the most satisfactory:

| | |
|---|---|
| Fel. bovis ins., gr. ii..... | To assist and strengthen the liver. |
| Ext. pancreatin, gr. ii... | To aid digestion and increase the secretions. |
| Caffeine muriate, gr. i... | To improve circulation and stimulate elimination. |
| Colocynth, 1-30 gr...... | For constipation. |
| Cocaine hydrochlorate, ½ gr., or ext. opii, 1-6 gr., Merck's...... | If severe depression. |

The diet must be regulated, and I usually eliminate potatoes, as the starch in potatoes oxidises so rapidly, and use rice or macaroni instead. An excellent daily diet is:

Two eggs, one-half to one pound of meat, one quart of milk, seven ounces of bread. Let me illustrate by a case or two.

Case I.—Mr. L. G., aged 52, German; occupation, engineer: family history negative. For the past ten years, at irregular intervals, he has had attacks of depression preceded by loss of appetite, indigestion, constipation, diminished elimination of urine, coated tongue, foul breath and vertigo, and later insomnia; also irritability, so pronounced that his family could hardly endure his presence in the home,—these very severe attacks of depression were accompanied by weeping. He lost from 20 to 30 pounds of flesh; felt cold and weak. The temperature averaged 98°, pulse, 58; urine scanty, deep colored and 1030 specific gravity. Urea, ½ per cent.; indican, marked. Uric acid crystals and calcium oxalates in great number. This is the usual history of his attacks with some slight variation in symptom. The treatment given him is already outlined elsewhere in this paper, and has restored the patient to normal health when suffering from his various relapses.

Case II.—W. W. C., aged 11. Family history, negative; had passed through some of the diseases of childhood without sequelæ. He was brought to me with the following history: great fear at night and if left alone during the day; marked irritability and depression; over sensitive and felt that none of his family loved him; cried frequently and was greatly depressed. He was discovered walking toward an incoming train on the railroad track, and in explanation said he "was going to let the train kill him, he felt so badly." This undoubtedly was a case of melancholia induced by toxemia. I found on inquiry and examination, a quick pulse, heavily coated tongue, fetid breath, choreic manifestation of the facial muscles, throat and nose; constipation, and the urine loaded with the uric acid; indican, oxalates, high specific gravity and low urea; no albumin or sugar. Purgatives, eliminatives, digestives and antifermentatives with frequent baths, controlled this attack in a few weeks, and in over four years there has been no return, as his diet and habits are carefully observed by his family.

Many of these cases are of a few days duration and easily controlled, but where we have chronic toxemia from faulty elimination and digestion, the condition is often most trying. We recall the irritating influences in the nervous system of carbonic acid gas, uremia, and diabetes, and many mental disturbances are reported, giving these influences as their probable exciting causes. Among the toxic insanities we also include alcoholism, which, by the way, is the most frequently met with by a large per cent.; also morphinism, cocainism, metal poisoning, and occasionally he suffers from excessive cigarette smoking.

Savage says, "Alcohol acts directly upon the brain as a poison; it acts indirectly upon the brain by impairing nutrition and interfering with the depuration of the blood." We have observed that large single doses of stimulants may act almost like a shock and render the person taking them powerless, or in some cases suddenly maniacal. In chronic drinkers the whole nutrition of the body suffers. There is a progressive loss of mental power which resembles in many particulars progressive paralysis of the insane. Repeated attacks of delirium tremens shock the nervous system severely, often developing acute mania or dementia. The detrimental influence of alcohol is greatly increased, if there be strong neurotic inheritance.

The pathological changes in subjects addicted to alcohol, according to Gowers, may be looked upon as hardening or sclerosis of the nerve tissues, so that conduction of impressions is retarded and the impressions themselves are imperfectly received and slowly organised. Statistics give chronic alcoholism as the most frequent cause of criminal insanity, and it is a factor producing great aggravation of the symptoms or manifestations observed in the various insanities, either functional or organic. Polyneuritis, tremors, epilepsy, and hysterical manifestations are often observed with or preceding alcoholic insanity. The prognosis in acute alcoholic insanity is good, except that we usually have a neuropathic basis which accounts for the frequent relapses. Plumbism and other metal and drug poisonings, producing nervous and mental disturbances, are observed and written of at some length. Those of the first class, plumbism, present a great deal of motor disturbance; the drop wrist, incoördination, painful neuritis and cachexia, with mental changes, suggesting paretic dementia. It is stated that the irritation and degeneration of the cell fibers, arteriosclerosis and gliosis of syphilis, may be due to toxins created by specific microörganisms. Of the acute infectious diseases, causing or preceding mental disease or psychoses, la grippe, typhoid fever, malaria, pneumonia, acute rheumatism, and neuritis doubtless head the list.

In private practice, and in all of our large institutions for the insane, we very frequently find la grippe given as the first cause of insanity. Mental symptoms often develop acutely with general grippe symptoms; again the mental disturbance may be delayed for weeks and develop apparently from a condition of chronic toxemia infection or exhaustion. In other cases I have seen, the mental collapse had been preceded by grippe, and then rheumatism or pneumonia. These are trying cases of long duration and fill one with anxiety. Some such cases under my care have had apparently normal intervals, but on recovery the entire period seems to be a blank or filled with confusion, delusions and morbid fears. Their minds often remain weak for a long period after all mental distress has disappeared; they are easily fatigued, weep and have a tendency to sleep a great deal, showing but slow recovery from their exhaustion and autointoxication. Recently a case of cervico-brachial neuritis with most persistent mental derangement and accompanied by general inflammatory rheumatism came under my care; the mental symptoms continuing for weeks after all fever and pain had ceased.

I am surprised at finding so little written on the subject in medical books and journals, for the condition is quite frequently met with and has been a source of great anxiety to many physicians who feared the mental disturbance would become a chronic condition. The term, post febrile insanity is given to disorders which complicate the crisis or convalescent period of acute fevers, as malarial, scarlet, and typhoid fever. Illusions, hallucinations, delusions of identity and great anxiety are the early mental manifestations. Pleasurable delusions or ideas of grandeur often develop. Most of these cases recover rapidly, continuing rarely more than a few weeks. In some cases, however, a more chronic course is observed, and there is a marked stupidity and confusion of ideas throughout the illness. Malarial fever is sometimes accompanied by mental disturbances with lucid intervals corresponding to the period between the attacks.

Case III.—Mrs. B. came under my care a few months ago, during her convalescence from typhoid fever. I was told the fever had been of four weeks' duration and she was making a good recovery, when she developed delusions and illusions. At times they were terrifying in character; again she was abnormally gay and happy. Insomnia was a very obstinate symptom. The disturbed periods continued only for four weeks, and in three months recovery was practically complete, except that her memory was weakened, but this was ultimately restored to normal strength.

Several cases of shorter duration have come under my observation, complicating or following measles and pneumonia, when the mental disturbance was less trying, resembling a condition of melancholy and weak-mindedness, or slight excitement, the chief anxiety with the family being that the patient might drift into dementia or chronic insanity. Whether engaged in general practice or doing the work of an alienist and neurologist, we are all constantly meeting this line of cases and it is well to have the ideas presented here in mind, thus making our diagnosis and plans for action clearer, and perhaps saving many patients from drifting into chronic organic mental diseases; also, preventing patients from being committed to institutions for the insane, when a few weeks of skilful patient treatment at home, or in a general hospital, might restore them physically and mentally to normal health.

BIBLIOGRAPHY.

Wagner, February, 1903, Klinische Wochenschrift.
F. Mott, from London Lancet, 1901.
Macpherson, London Lancet, nervous diseases.
Franz Glenard, Le Progres Medical, March, 1902.
Savage, On insanity.
Gowers, Nervous diseases.
Peterson: Peterson & Church, Nervous and mental diseases.

429–431 University Block.

DISCUSSION.

Dr. E. H. Howard, Rochester: Permit me to utter a word relative to this very interesting and important paper, merely to accentuate its importance. At a recent meeting of the American Association of Superintendents of Hospitals for the care of the insane a whole day was given up to the consideration of the first portion of this paper—namely, toxins as causative factors in insanity. We were edified and instructed by a series of papers from various parts of the United States and Canada, widely separated sections, where studies and efforts were made in this relation, and were told a great deal about test meals, the effect of hydrochloric acid on the stomach, and of a group of cases where there was an excess of this element. A great deal of time was taken by the physiologists and students in the chemical laboratories in the hospitals, in trying to understand these cases.

Dr. Hill, of Baltimore, in discussion, said: "A general practitioner hasn't time for all this test meal business, hasn't the apparatus or the inclination, and I find I get on well by a little experimentation. When a patient comes into my office with these mental symptoms, I look upon it as probably a case of this character, and prescribe bicarbonate of soda, directing him to come back in three days. If the patient is not better, showing a marked degree of improvement, then I prescribe hydrochloric acid and find the cause of the trouble." Dr. Hill even made a little sport of the doctors who exploited their laboratory work with the test meals.

As to that portion of the paper, it is very well, indeed, to be reminded that we must be alert to this possibility, because it must make a difference sometimes in the care of the infectious disease. I wish to express my appreciation of the excellent paper, and incidentally, without attempting to discuss the president's address, I may add that his remarks about disinclination to send patients to hospitals for the insane meets the hearty approval of the hospital superintendents. It is a great surprise to many of us that so many patients are sent so hastily to hospitals for the insane.

Dr. F. S. Crego, Buffalo: I desire to add a word concerning the advisability of keeping these patients at home. I am a strong advocate of keeping patients with mental disease at home, and also I think the profession at large should learn something about insanity. In my course of lectures at the University of Buffalo every year I try to teach the students a few preliminary and primary ideas of insanity, but, as a rule, they do not like to attend lectures on this subject.

We have had struggles with almost every class to get them to attend, and now we put a few questions into the examination papers with the object of compelling attendance, but they seem to learn but very little. They do not study the subject enough to judge of a case, and yet they will sign a paper of commitment.

not for the actual money but for the relief, as they suppose, of the patient. I am of the opinion that 50 per cent. of the patients sent to the state hospitals are cases that should not go there. Indeed, if the truth were really known, there is a large number of patients sent to state hospitals every year that are not insane, but are cases of typhoid or toxemic delirium.

Not long ago I was called to testify in the southern part of the state in a murder trial, in the course of which one of my students testified on the other side. When asked what his experience was, he said he had heard Professor Crego's lectures and also been to New York. He said he knew all I knew and all the New York men knew. After he left the witness box I asked him how many of my lectures he attended, happening to remember he was marked for not attending. He attended two lectures and I think one clinic which comprised his instruction on the subject of his testimony.

Dr. R. G. Cook, Rochester: The president's address and Dr. Stephenson's paper both emphasise the need of a pavilion for the care of mental and nervous cases. We have been working for one in Rochester a good while, thus far without success, but we must not despair. Dr. Stephenson's paper is interesting, instructive, and valuable, and in only one point would I disagree with him—namely, in regard to the recognition of toxins in recent years. We are apt to think that it is only the modern writers that have recognised these conditions. Now, as a matter of fact, in the old days when physicians believed in humeral pathology, they described clinical conditions, which correspond exactly to some conditions which we recognise now; one, for instance, is lithemia. Recently in looking over a book on insanity written by Dr. Pritchard, in 1835, I discovered that he gave an exact description of the lithemic condition as one of the exciting causes of insanity, and the treatment was not very different from that which we now use.

Another point about the care of these cases to be borne in mind is in regard to prognosis. These patients recover from their individual attacks, but a person that becomes delirious or sinks into a condition of stupor from a comparatively mild toxemia, is one who is always in danger of becoming insane from very slight exciting causes. This was strongly emphasised in a case I happened to meet in my college days. A young man who was either very moody or very jolly, one or two glasses of beer or ale would make one of the merriest persons in college. We did not think there was anything the matter with him at the time, but he has spent over half the time since graduation in a hospital for the insane. This goes to show that individuals who show mental symptoms from a mild toxemia must be watched carefully when under our general care for mental symptoms on very slight exciting cause.

Dr. B. C. Loveland, Syracuse: In the first place, under the question of toxemia which causes mental disturbances, permit me to say there are two certain classes. In one class will be included those of intestinal origin; in the other class are cases which result from infectious fevers and the like. Another point is that in the mind of the general practitioner the idea of insanity as a disease has not taken hold. It is mysterious, it is beyond the conception of those who look for a real pathology or causation, hence it is impossible for a large number of physicians to grasp the truth in regard to mental observations.

It has been impossible, due to the lack of facilities, for the average practitioner and particularly for the student in many of our smaller colleges, to have an opportunity of studying the course of mental disease. The keeping of patients at home has been advocated today, which is a very valuable way of affording an opportunity to study the course of the disease. I believe it is desirable to keep patients who are not dangerous to themselves or others, or who can be well handled, at home as long as possible, giving them the best treatment that can be administered there, with the hope and expectation that it will be more efficacious than treatment at hospitals. The necessity, already referred to, for a detention ward or pavilion in every city of considerable size, should receive the serious consideration of every medical man. When such are established the profession can get such a knowledge of these cases as will be of use to it.

Dr. J. Henry Dowd, Buffalo: I was interested in Dr. Stephenson's paper, for one reason among others, that we do not know the day when these patients may reach the dangerous stage described. What I would like to know is, how can we tell just before they reach the turning point? In my work on the urethra and bladder, I have mentioned that it is as important to test for indican in the urine as for albumin, and I will say now, it is a good deal more important to test for indicanuria. A young woman was sent to my office to have an examination of the urine made, the diagnosis being optic neuritis. The examination was made especially for albumin, but there was none, though I found indicanuria. She had no symptoms of indicanuria or intestinal fermentation. She was put upon salicylate of soda in large doses. Another patient was a young woman, married about three years, but had never been pregnant. Her husband asked me to examine her urine, and upon doing so I found marked indicanuria. I administered salicylate of soda and nux vomica. The husband consulted me in about three weeks, saying his wife did not menstruate. I asked him to bring a specimen of urine in about three weeks more, which he did, and after an analysis of it I pronounced her pregnant. She was confined about eight months afterward. Both patients presented mental disturbance.

Dr. W. J. Herriman, Rochester: I have been very much interested in what has been said on this subject, and I wish particularly to emphasise Dr. Crego's remarks about the general practitioner's need of more knowledge on the subject of insanity, but, also, I think it is time for a word of warning lest errors be made in keeping dangerous patients at home. Dr. Crego tells us that 50 per cent. of the cases sent to state hospitals should not be sent there; while Dr. Howard tells us the superintendents of the state hospitals are surprised at the number of patients who are sent to those institutions that might have been kept at home. I agree fully with these gentlemen, that when a case is known (if it is possible to know such a thing) to be harmless to himself or to others, and there is a fair prospect that such a patient may be treated successfully at home, the case should be treated at home by all means; but I do believe that if more cases of a dangerous nature were sent to a hospital for care promptly, a great many lives, especially of women and children, would be saved that are sacrificed to the delusions of an insane person. How frequently we read in the newspapers of a woman who has killed her children, and, perhaps, herself; that for some time she has been acting strangely, and was supposed to be deranged but harmless. That woman should have been isolated in a proper hospital. She was not a case for calomel and hydrochloric acid. Such are cases for restraint, and I drop this little word of caution: don't wait too long with the cases of a suicidal or homicidal tendency, or they may commit crimes.

Dr. E. B. Potter, Rochester: I agree with Dr. Herriman in what he just said, and I am of the opinion that the general practitioner is apt to err on the wrong side in sending cases to the hospital. Dr. Howard and myself have worked together for twenty years, and we have always been careful with our statistics. I know of but two or three cases having been sent to us in that time which were not insane, easily demonstrable as such, and they were cases that were benefited by coming to us. The idea of a detention hospital is growing. We are working toward it steadily. It is proof that we are working toward it that so much time is spent in this meeting this morning discussing this topic. A few years ago the subject of insanity would not have taken up much time. This morning it has been interestingly discussed, which shows that Dr. Crego's lectures are doing good, and all centers of learning are now adding to their courses something on this topic, which is growing in importance. I know that the idea that insanity is a disease and not devilishness is becoming understood in the profession and by the people, showing that they are being educated on this subject. Half of the people who come to us now know that they are brought to the hospital for treatment. They come to us for treatment as they would for a broken leg or a tumor. They come because they are sick and need treatment.

A member[1]: There is one advantage in the hospital treatment which is superior to treatment in the home; that is the

1. This visitor was unknown to the chair and was not announced when he discussed the paper.—C. A. G., Secretary.

moral influence, as it might be designated. Oftentimes in their whims, their delusions, things are turned upside down to gratify the insane, and frequently that is the worst thing that can be done for the patient. A short experience in the state insane service impressed this lesson upon me—namely, that the very fact that their whims and notions and insane demands were largely ignored and simply their necessities administered to, had a most salutary influence upon the mental condition. I believe there was a small percentage whose recovery was simply due to the fact that they were put in a place where their every whim was not gratified.

Dr. William J. Howe, Scottsville: As a general practitioner from a country district, I am prompted to add that my experience has taught me that learned alienists are liable to err in their judgment as to the etiology of many of these mental conditions; at least, my experience has been such. A few years ago a strong, robust, young Irishman came to me, suffering with pronounced melancholia. He had been under the care of an eminent specialist who had treated him for nine weeks, with no improvement except as to his nutrition; he improved in flesh but mentally was no better. He came back to his country home, and I employed him as my coachman. I began an eliminative process to determine his condition or what was causing the melancholia. I found he had a double astigmatism. I stopped all medication at once. In a month, instead of sleeping one hour, he slept practically all night long; and, to shorten the story, he made a perfect recovery. He had been in the hands of eminent, skilful men, and yet they had overlooked the cause of the trouble; so it seems to me that in endeavoring to learn more of insanity we should endeavor to learn, first, more of the cause or causes of insanity.

In further illustration I may mention that a few days ago it was my misfortune to have a relative sent from a western city suffering from melancholia. She is a comely young woman, 26 years of age, who had been for two years affected with melancholia. Her physician was one of the most eminent in the west. He had treated her for two years for melancholia, but had never made an ocular investigation or local examination. He sent her east to the country for a rest. An examination showed her to have a marked ulceration of the os uteri, with a slight astigmatism in each eye, and considerable muscular tension. She has been in the country less than three weeks, and is decidely improved. The astigmatism has been corrected, the muscular tension has been somewhat modified, and she is improving with a fair prospect of recovery. In endeavoring to learn how to manage insanity let us learn, first, what the cause of each individual case is. It is a difficult matter to settle the treatment of a case unless we know the primary cause, so I would like to emphasise the importance of careful investigation as to the cause, and find out if possible what produces the mental symptoms, then remove the cause, and the rest will become easier to deal with.

Dr. A. A. Young, Newark: I wish to say one word in approval of Dr. Howe's remarks. Our books give us signs and symptoms of insanity; pathology and etiology largely are left out. We cannot sit and hear lectures fresh from the teacher. We have got to take patients as we find them. Friends say they are insane, and must be sent away. The ease with which we get them into our asylums is another element to take into consideration. Not a word ever comes back to us in regard to a patient's insanity, whether he is insane or not. The feelings of practitioners should never be taken into account, but if patients are not insane the superintendents of asylums should send them back promptly and so pronounce them. I find also another trouble: we cannot get from our asylums the information we want as to when the patient is about to cross the line from sanity to insanity. I plead for more information on the etiology and pathology of insanity and less on the technic of management. Give that to us and then we will send less to asylums.

Dr. Crego: I desire to correct a possible wrong impression that my remarks may have caused. In the first place, I do not advocate keeping dangerous homicidal patients at home; I do not advocate keeping suicidal patients at home unless they are under strict guard. What I am advocating is that the general practitioner should study their cases with care, and then they will recognise the man that is going to kill his children, and the woman who is dangerous, and they will not keep them at home for a minute, but will put them in a proper place of detention. It is unnecessary to keep those cases at home, it is not right to do so. In the next place, they should not be put in a room by themselves and allowed to go without treatment. They need treatment the same as a man with pneumonia or typhoid fever. It is a disease they suffer from and they are not going to dream it away; it is not going to disappear unless the doctor gives them some relief. Dr. Howe has hit the nail on the head; we must get at the cause and pathological condition,—pathology and etiology. I think the new books on insanity are pretty profuse and prolific on these points.

Dr. J. P. Creveling, Auburn: I would like to say one word in support of Dr. Crego's first remark, that the student of today does not study insanity or attend the lectures on that subject. I believe that for the last four or five years the question has appeared in the questions put by the state examining board every year, "Define an illusion, a delusion, and a hallucination." Some of the most absurd answers have been given. As an illustration I give you one. A candidate, in describing an illusion, said it was "a belief a man has that he is God, when he isn't." Though this man was from Buffalo, I don't believe he ever attended Dr.

Crego's lectures or he would not have made such an answer. I heard only the last part of Dr. Stephenson's paper, where he paid a great deal of attention to diseases of fermentation, as I infer, of the stomach. It is my impression that more toxic conditions follow from a clogged colon than from a fermented stomach. Another class of cases where there has been a good deal of mental disturbance—not insanity—has come under my care which have been associated with a gouty condition, and I believe in many instances the cause of mental aberration may be traced to gout and, likewise, to kidney lesions that have not been recognised.

Dr. Stephenson: I have nothing to add. I believe the success of any paper must be admitted from the discussion which it has elicited, and as this has been going on for more than an hour, I think that I can congratulate myself. I thank the gentlemen very much for responding so liberally in the discussion.

